# Prophylactic treatment of hyperbaric oxygen treatment mitigates inflammatory response via mitochondria transfer

**DOI:** 10.1111/cns.13124

**Published:** 2019-04-11

**Authors:** Trenton Lippert, Cesario V. Borlongan

**Affiliations:** ^1^ Department of Neurosurgery and Brain Repair, Center of Excellence for Aging and Brain Repair USF Morsani College of Medicine Tampa Florida; ^2^ University of South Florida Honors College Tampa Florida

**Keywords:** hyperbaric, mitochondria transfer, preconditioning, stroke, traumatic brain injury

## Abstract

**Aims:**

Hyperbaric oxygen therapy (HBOT) has been widely used as postinjury treatment; however, we investigate its ability to mitigate potential damage as a preconditioning option. Here, we tested the hypothesis that HBOT preconditioning mitigates cell death in primary rat neuronal cells (PRNCs) through the transfer of mitochondria from astrocytes.

**Methods:**

Primary rat neuronal cells were subjected to a 90‐minute HBOT treatment at 2.5 absolute atmospheres prior to either tumor necrosis factor‐alpha (TNF‐alpha) or lipopolysaccharide (LPS) injury to simulate the inflammation‐plagued secondary cell death associated with stroke and traumatic brain injury (TBI). After incubation with TNF‐alpha or LPS, the cell viability of each group was examined.

**Results:**

There was a significant increase of cell viability accompanied by mitochondrial transfer in the injury groups that received HBOT preconditioning compared to the injury alone groups (44 ± 5.2 vs 68 ± 4.48, n = 20, *P* < 0.05). The transfer of mitochondria directly after HBOT treatment was visualized by capturing images in 5‐minute intervals, which revealed that the robust transfer of mitochondria begins soon after HBOT and persisted throughout the treatment.

**Conclusion:**

This study shows that HBOT preconditioning stands as a robust prophylactic treatment for sequestration of inflammation inherent in stroke and TBI, possibly facilitating the transfer of resilient mitochondria from astrocytes to inflammation‐susceptible neuronal cells in mitigating cell death.

## 1 INTRODUCTION

Central nervous systems (CNS) diseases encompass a wide variety of pathologies.^1^ However, stroke and traumatic brain injury (TBI) are the most prevalent neurodegenerative diseases of adult‐brain in the United States, which can strike a wide spectrum of people.^1^ According to the American Heart Association, stroke is the fifth‐leading cause of death in the United States, killing approximately 130 000 each year. The associated healthcare costs for stroke exceed $33 billion per year. Stroke is the leading cause of long‐term disability in the United States.^2^ Comparably, in 2013, TBI resulted in 2.8 million emergency room visits, hospitalizations, and deaths, accompanying the approximately 3.1 million people living with TBI‐related diseases, with an associated healthcare cost of $76.5 billion in 2012.^3^, ^4^ Stroke and TBI share some similar pathologies regarding the primary and secondary cell death mechanism largely resulting from chronic neuroinflammation.^5^ A key common pathological feature is the formation of a necrotic tissue core, which is unrecoverable, following stroke and TBI.^6^, ^7^, ^8^ The onset and progression of secondary cell death of both diseases has been linked to the blood‐brain barrier (BBB) breakdown, allowing various inflammatory cytokines to permeate the BBB, infiltrate the brain, and upregulate the inflammatory response,^9^ altogether worsening the disease outcomes. In addition, several other exacerbating factors, such as oxidative stress, apoptosis, and mitochondrial dysfunction, have been shown to contribute to additional neurodegeneration following BBB damage.^10^, ^11^, ^12^, ^13^

Hyperbaric oxygen therapy (HBOT) has been a treatment of interest for stroke as well as TBI over the past decade.^14^, ^15^ HBOT utilizes a pressurized chamber, usually 2‐3 absolute atmospheres (ATA), resulting in hyperoxygenation of tissues, inducing local angiogenesis in damaged regions of the body and recruitment of progenitor cells to the damaged regions.^16^, ^17^, ^18^ The common, FDA‐approved, uses of HBOT include treating patients with open wounds, often resulting from burn injuries or diabetic ulcers.^19^, ^20^ The ability for a patient to undergo HBOT during the acute stage of stroke has been difficult to accomplish due to the overall timeline of the ischemic event and a limited number of facilities having the necessary equipment; therefore, the chronic stages of stroke have been targeted as a possible therapeutic window for HBOT.^14^ This regimen seeks to ameliorate cognitive impairments synonymous with stroke, such as memory loss, language and comprehension deficits.^14^, ^21^ Similarly, the unforeseen occurrence of TBI presents as a logistical hurdle in introducing HBOT as an acute treatment regimen for brain trauma patients.^22^ Mechanistically, the secondary cell death with wider therapeutic window, characterized by inflammation has become the main target of HBOT treatment research, as reducing the levels of inflammatory cytokines has been linked to limiting peri‐infarct/peri‐impact tissue loss.^23^, ^24^ However, the therapeutic mechanism of HBOT mediating the sequestration of inflammation is not fully understood.^25^

A key unexplored research theme in the use of HBOT as a treatment for stroke and TBI involves investigations into the role of the preconditioning paradigm. Several studies have shown the preclinical efficacy of HBOT preconditioning for attenuating neuronal cell loss following an ischemic or traumatic event, but little mechanism‐based assessment has elucidated the therapeutic pathways solicited by HBOT in these studies.^26^, ^27^, ^28^, ^29^ The importance of alternative therapies for stroke and TBI patients has become evident.^30^ To this end, cognizant that mitochondrial dysfunction closely approximates a pathologic inflammatory response resulting from ischemia and traumatic insult, evaluation of the mitochondria poses as a logical target of investigation in order to begin to understand the mechanism of HBOT preconditioning.^31^, ^32^, ^33^ Of note, healthy extracellular mitochondria have been demonstrated to transfer from astrocytes into neurons following stroke resulting in reduced neuronal cell death.^34^

In this study, we explored the ability of HBOT preconditioning to limit neuronal cell death following inflammatory insults, mimicking the secondary cell death associated with ischemic stroke and TBI. More importantly, the potential role of HBOT in transferring mitochondria as a therapeutic mechanism in facilitating survival of the neuronal cells was also examined.^35^ We hypothesized that a transfer of astrocytic mitochondria into neurons, following HBOT preconditioning, would improve neuronal cell viability following inflammatory insults.

## 2 MATERIALS AND METHODS

### 2.1 Cell culture

**Figure 1:**
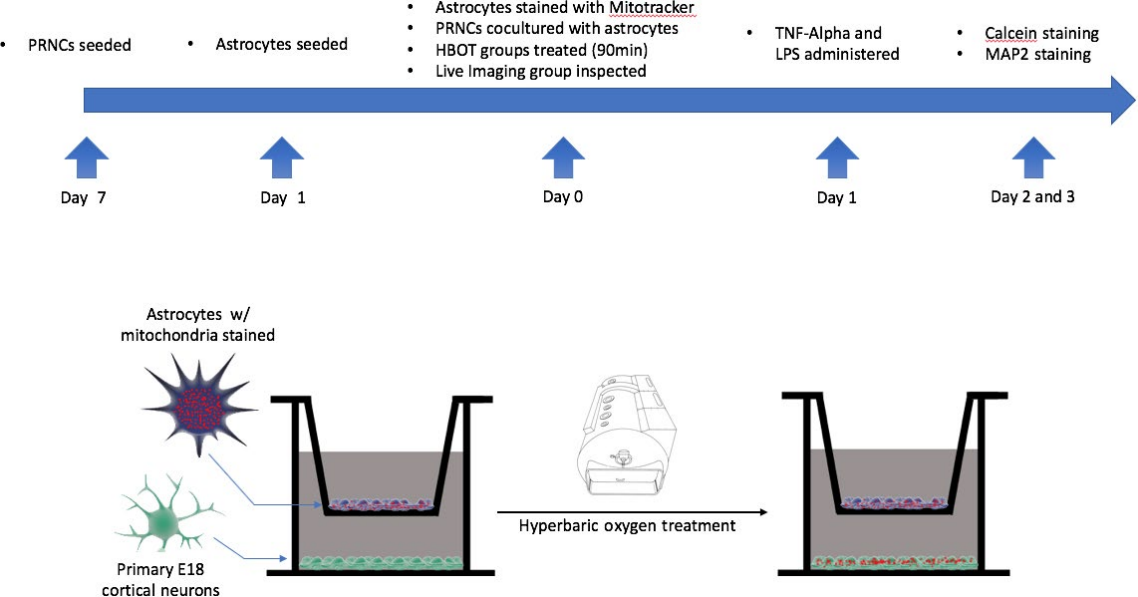
Experimental design. Timeline of experiment, including cell culture and immunocytochemistry. Hyperbaric oxygen preconditioning in vitro treatment. Astrocytes stained with Mitotracker are cocultured on top of E18 primary cortical neurons (PRNCs) in a growth medium. The cocultured plates were then placed in a hyperbaric oxygen chamber for treatment. Mitotracker of astrocytes was found in the primary E18 cortical neurons

The research procedures involving animals were approved by the USF Institutional Animal Care and Use Committee (IACUC). Primary rat neuronal cells (PRNCs) were dissected from cerebral cortices of E18 Sprague‐Dawley rat embryos. Cells were seeded on poly‐D‐lysine (Fisher Scientific, ICN10269480) coated glass coverslips in 6‐well plates (Fisher Scientific, 0877124) and cultured in Dulbecco's Modified Eagle Media (DMEM, Fisher Scientific, 10567‐014) containing 4.5 g/L glucose, l‐glutamine, 25 mmol/L HEPES, 10% fetal bovine serum (FBS, Fisher Scientific, SH3007103) and 1% antibiotic/antimycotic at a density of 1.5 × 10^6^ cells per well. After 24 hours, the medium was changed to Neurobasal medium (Fisher Scientific, 21103049), supplemented with B‐27 (Fisher Scientific, 17504044). Cells were cultured in incubator at 37°C with 5% CO_2_. Cells were utilized for experiments 7 days after seeding. Astrocytes derived from U‐87 MG Astrocyte Cell Line (Sigma, 89081402). The cells were passaged six times for growth in T‐175 flasks (Fisher Scientific, 12562001) in DMEM with 10% FBS and 1% penicillin/streptomycin (Fisher Scientific, 15140122). One day prior to coculture, the astrocytes were seeded on a 0.4 μm mesh plate insert (Fisher Scientific, 0877115) in DMEM with 10% FBS and 1% penicillin/streptomycin at a density of 3 × 10^5^ cells per well. On day 7 postseeding, the astrocyte mesh inserts were placed in coculture with the PRNCs (Figure 1).

### 2.2 HBOT

**Figure 2:**
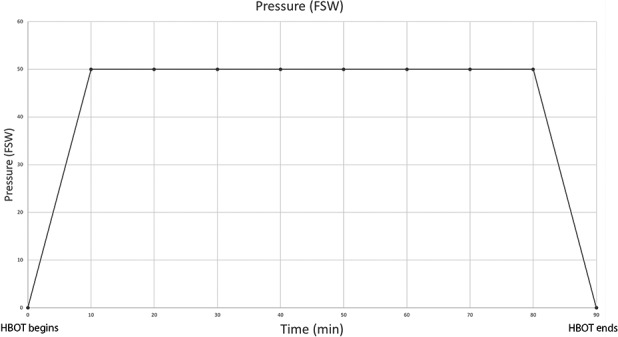
Hyperbaric oxygen therapy course. Pressure graph of hyperbaric oxygen preconditioning. During the first 10 min, there is a constant increase of pressure until the chamber reaches 49.5 feet of seawater (FSW). This pressure is sustained for 70‐min, followed by a constant depressurization of the chamber over a 10‐min period. The total length of the preconditioning treatment is 90 min

Hyperbaric oxygen therapy was administered using an OxyCure 3000 hyperbaric incubator (OxyHeal Health Group, National City, CA, USA). The HBOT regimen consisted of a single treatment at 2.5 absolute atmospheres lasting 90 minutes, with a 10‐minute ascent and descent period (Figure 2). Following HBOT the cultures were returned to an incubator at 37°C and 5% CO_2_. Cultures remained in incubation for 24 hours prior to insult.

### 2.3 Injury

To recreate the secondary cell death of inflammation observed in stroke and TBI, we employed two established inflammation‐inducing agents.^36^, ^37^, ^38^ The tumor necrosis factor‐alpha (TNF‐alpha, Fisher Scientific, 210TA020CF) only and HBOT plus TNF‐alpha cocultures were treated with 50 ng/mL of TNF‐alpha (RD) for 24 hours^39^ in the incubator at 37°C and 5% CO_2_. The Lipopolysaccharide (LPS, Fisher Scientific, NC0202558) injury and HBOT plus LPS groups were treated with 100 ng/mL of LPS and returned to the incubator.^40^ Each insult used the same medium as previously described for neurons and astrocytes. The control plates remained untreated.

### 2.4 Measurement of cell viability

After the 24‐hour incubation with TNF‐alpha and LPS, all groups were assessed for cell viability using calcein‐AM (Fisher Scientific, 4892010K). After treatment, the astrocyte mesh inserts were removed and the remaining 6‐well dish was incubated with 1 μmol/L calcein‐AM for 30 minutes in the incubator at 37°C with 5% CO_2_. Bright green fluorescence was retained within living cells. Five images were captured per well in randomly selected regions to determine cell viability at 10×. Analysis of cell intensity was performed by ImageJ (NIH). During cell counting, 30.25 cm^2^ regions were counted using ImageJ and calculated to determine the number of cells per 1 cm^2^. Analysis of data was conducted using GraphPad Prism 6 (GraphPad Software, La Jolla California USA).

### 2.5 Immunocytochemistry

Prior to coculturing neurons and astrocytes, each was labeled with mitotracker, in order to visualize mitochondria belonging to each cell type. Astrocytes were labeled with Mitotracker Deep Red FM (Fisher Scientific, M22426) for 30 minutes in DMEM with 10% FBS and 1% penicillin/streptomycin (Fisher Scientific, 15‐140‐122) with 500 nmol/L of Mitotracker Deep Red. Neurons were labeled with Mitotracker Green FM (Fisher Scientific, M7514) for 30 minutes in Neurobasal with B‐27 supplement at 37°C and 5% CO_2_. Following HBOT and insult, cells were rinsed with Dulbecco's phosphate‐buffered saline with calcium and magnesium (DPBS, Fisher Scientific, 14080055) and then fixed with 4% paraformaldehyde (PFA) for 20 minutes at room temperature. Cells were rinsed again with DPBS. Cultures were permeabilized with 0.3% Triton‐X for 5 minutes at room temperature. Plates were rinsed with DPBS before blocking with 5% goat serum at room temperature. Mouse anti‐MAP2 1 μg/mL was added to each well and incubated overnight at 4°C. After rinsing several times with DPBS, Alexa Fluor 488 goat anti‐mouse 1 μg/mL was added and incubated for 60 minutes at room temperature. Plates were then rinsed with DPBS, and coverslips were placed on slides using Vectashield with 4,6‐diamidino‐2‐phenylindole DAPI (Fisher Scientific, NC9524612). Immunostaining images were captured using an Olympus FV1200 Spectral Inverted Laser Scanning Confocal Microscope. Colocalizations of astrocytic mitochondria with PRNCs as well as with endogenous mitochondria from PRNCs were assessed.

### 2.6 Statistics

The data were evaluated using ANOVA followed by post hoc Bonferroni's tests. Statistical significance was preset at *P* < 0.05. Data are presented as mean ± SE from quintuplicates of each treatment condition.

## 3 RESULTS

### 3.1 Hyperbaric oxygen treatment rescues cell viability

**Figure 3:**
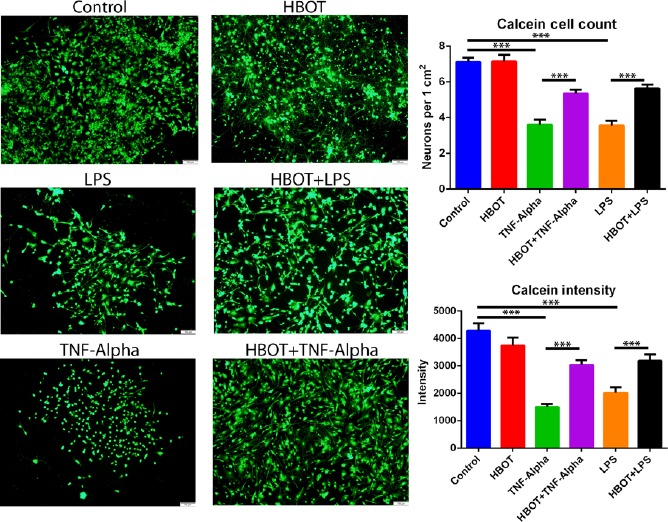
Primary cortical neuron cell viability. Administration of Hyperbaric oxygen therapy (HBOT) precondition occurred 24 h prior to injury onset. Primary rat neuronal cells (PRNCs) cocultured with astrocytes were subjected to HBOT for 90 min in the preconditioning group. After the insult was introduced to the PRNCs cocultured with astrocytes for 24 h, the cell viability was analyzed by Calcein‐AM staining. Cell counting was performed and standardized per 1 cm^2^. Intensity of signal was also calculated as described in the materials and methods. *P < 0.05, **P < 0.01, ***P < 0.001. The scale bar corresponds to 20 μm

Calcein‐AM cell viability staining was performed and imaged using a fluorescent inverted microscope (Figure 3). Cell viability of E18 cortical neurons cocultured with U‐87 astrocytes was measured via cell counting of neurons and intensity of calcein staining. Control E18 cortical neurons had an average of 7.11 cells per 1 cm^2^ and an average intensity of 4290. E18 cortical neurons exposed only to HBOT had an average of 7.15 cells per 1 cm^2^ and an average intensity of 3744. Cortical neurons administered TNF‐alpha had an average of 5.60 cells per 1 cm^2^ and an average intensity of 1507, while cortical neurons administered LPS had an average of 3.56 cells per 1 cm^2^ and an average intensity of 2025. The cortical neurons exposed to HBOT and then administered TNF‐alpha had an average of 5.34 cells per 1 cm^2^ and an average intensity of 3045. The cortical neurons exposed to HBOT and then received LPS had an average of 5.64 cells per 1 cm^2^ and an average intensity of 3192. A One‐way ANOVA was conducted to analyze the effect of HBOT on each condition. There was a statistically significant effect of HBOT on viable cell count per 1 cm^2^ for all six conditions (*F*_5,167_ = 33.18, *P* < 0.001). There was also a significant effect of HBOT on cell intensity for all six conditions (*F*_5,182_ = 22.29, *P* < 0.001).

Post hoc comparisons using Bonferroni's tests indicated the mean number of cells per 1 cm^2^ for the control group was not significantly different than the HBOT group. However, the TNF‐alpha and LPS groups showed a significant reduction when compared to the control group (*P* < 0.001, *P* < 0.001). HBOT preconditioning prior to TNF‐alpha insult resulted in a significant increase in cell bodies when compared to the TNF‐alpha group (*P* < 0.001). Similarly, the HBOT + LPS group demonstrated a significant increase in viable cell bodies versus the LPS group (*P* < 0.001).

Post hoc comparisons using Bonferroni's tests were also conducted for cell intensity. These results reiterated there was no significant difference in the means between the control group and the HBOT preconditioning group. Again, there was a significant reduction of intensity in the TNF‐alpha and LPS groups when compared to the control group (*P* < 0.001, *P* < 0.001). When HBOT was administered prior to TNF‐alpha it yielded a significantly increased intensity versus the TNF‐alpha group (*P* < 0.001). Also, HBOT preconditioning prior to LPS administration also resulted in a significant increase in intensity versus the LPS group (*P* < 0.01).

### 3.2 Hyperbaric oxygen therapy stimulates mitochondria transfer

**Figure 4:**
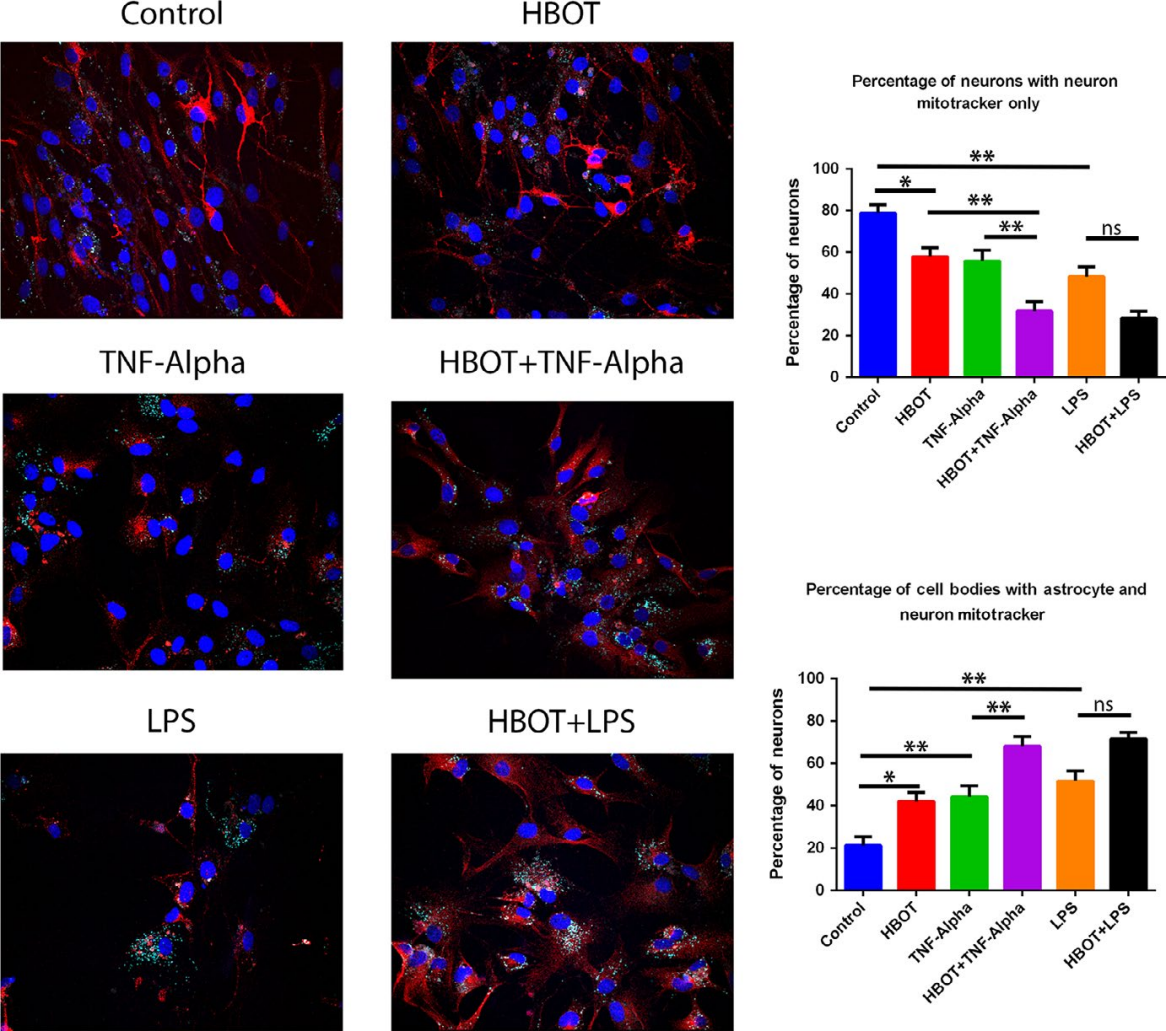
Mitochondrial transfer to primary cortical neurons. Migration of astrocyte mitochondria was tracked using Mitotracker staining of astrocytes prior to coculture with primary rat neuronal cells (PRNCs). Cocultures were subjected to the same preconditioning setting as previously described. Transfer of astrocyte mitochondria (cyan) was quantified by counting the number PRNCs containing cyan and dividing by the total number of PRNCs marked with MAP2 (red). DAPI was utilized to mark PRNC nuclei. *P < 0.05. ** P < 0.01. The scale bar corresponds to 20 μm

Mitochondria transfer was imaged using a scanning laser confocal microscope (Figure 4). The percentage of neuronal cell bodies containing astrocyte mitochondria was analyzed. The control cortical neurons demonstrated an average of 21.25% of neuronal cell bodies containing astrocytic mitochondria. The HBOT group which received only HBOT preconditioning and no insult had an average of 42.17% of neuronal cell bodies containing astrocytic mitochondria. Similarly, neurons that received TNF‐alpha and LPS treatment had an average of 44.23% and 51.71% of neuronal cell bodies containing astrocytic mitochondria, respectively. Cortical neurons exposed to HBOT preconditioning prior to TNF‐alpha insult had an average of 68.21% of neuronal cell bodies containing astrocytic mitochondria, while neurons exposed to HBOT prior to LPS insult had an average of 71.52% of neuronal cell bodies containing astrocytic mitochondria. There was a statistically significant effect of HBOT on the percentage of astrocyte mitochondria in neuronal cells for all six conditions (*F*_5, 103_ = 19.32, *P* < 0.001).

Post hoc comparisons using Bonferroni's tests indicated the average percentage of neuronal cells containing astrocytic mitochondria for the control group was significantly less than the HBOT preconditioning only group (*P* < 0.05). However, there was no significant difference between the HBOT preconditioning only group with the TNF‐alpha and LPS groups. HBOT preconditioning prior to TNF‐alpha insult resulted in a significant increase in astrocytic mitochondria presence in neuronal cell bodies when compared to the TNF‐alpha group (*P* < 0.01). Although the HBOT + LPS group failed to reach statistical significance compared to the LPS group, it displays a similar trend to the TNF‐alpha and TNF‐alpha HBOT preconditioning comparison (Figure 4).

### 3.3 Live imaging following HBOT demonstrates mitochondrial transfer

**Figure 5:**
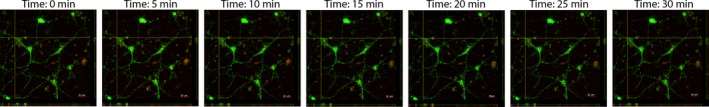
Live imaging of primary cortical neurons undergoing mitochondrial transfer. Rat E18 neuronal cells were harvested and seeded in poly‐D‐lysine coated (100 μg/mL) 6‐well plates at 1.5 × 10^6^ cells/well in Dulbecco's Modified Eagle Media high glucose with 1% antibiotic/antimycotic for 24 h. The media was changed every 3 d, and the cells were subcultured at 90% confluency as needed. Twenty‐four hours prior to the preconditioning, U87 astrocytes were stained with MitoTracker Deep Red FM (500 nmol/L) according to manufacturer's protocol and seeded into coculture inserts at 0.5 × 10^6^ cells/well. On the day of the experiment, rat E18 neuronal cells were stained with MitoTracker Green FM (200 nmol/L) according to manufacturer's protocol. The neuronal cells were then cocultured with U87 astrocytes for 3 h prior to Hyperbaric oxygen therapy (HBOT) administration. The cells were subjected to 70 min of HBOT at 2.5 ATA with 10 min pressurization and depressurization at a rate of 0.07 atm/min for a total of 90 min. Directly following HBOT treatment, the cocultured astrocytes were removed and the confocal z‐stacks live images were captured at 180× every 5 min for 30 min. Primary rat neuronal cell (PRNC) mitochondria: Green; Astrocyte mitochondria: Red. The scale bar corresponds to 20 μm

Live imaging was conducted using a scanning laser confocal microscope to capture images at 5‐minute intervals over a 30‐minute period (Figure 5). At acute period after HBOT initiation, live imaging revealed absence of Mitotracker‐labeled mitochondria at 0 minute but its presence at 5 minutes (Figure 5). Similarly, at delayed period following HBOT initiation between 15 minutes and 20 minutes, migration of Mitotracker‐labeled astrocytic mitochondria was visualized (Figure 5).

## 4 DISCUSSION

The present study demonstrated the therapeutic effects of HBOT preconditioning in protecting against the secondary cell death associated with cerebrovascular events, specifically stroke, and TBI. Primary neurons that were exposed to HBOT at 24 hours prior to an inflammatory insult exhibited a significant reduction in cell death compared to the injury‐only groups. Further analysis revealed substantial increase of astrocytic mitochondria in the inflammation‐insulted primary neurons, particularly in the HBOT‐preconditioned groups. Altogether, these results suggest HBOT reduces the deleterious inflammatory response, potentially through the transfer of mitochondria from astrocytes to neurons highlighting a highly innovative mechanistic pathway mediating the therapeutic efficacy of HBOT preconditioning.

Mitochondrial dysfunction stands as a therapeutic target in stroke and TBI due to its role in the secondary injury mechanism.^41^, ^42^ However, HBOT as a treatment for cerebrovascular diseases has yielded mixed results.^14^ The initiation of HBOT may dictate the therapeutic, or detrimental, outcomes. To date, the modality of HBOT preconditioning has been largely neglected; HBOT's use as a prophylactic treatment for cerebrovascular diseases has only recently come into the spotlight for stroke and TBI research. Recent studies have postulated various mechanisms underlying HBOT's neuroprotective effects, including stabilizing the BBB and reducing inflammation.^43^, ^44^, ^45^, ^46^ That BBB breakdown and inflammatory response are closely associated with mitochondrial impairment provided the impetus in the present study to examine the role of mitochondria as a therapeutic target of HBOT.^47^, ^48^ Indeed, the transfer of mitochondria from astrocytes into neurons was observed after stroke.^34^ Here, we showed that astrocytic mitochondria also transferred to neurons under ambient condition or when exposed to an inflammatory insult, but such transfer was more robustly recognized when treated with HBOT prophylactically. These findings form the basis for prophylactic HBOT for individuals who are at high risk of cerebrovascular events, specifically stroke and TBI, providing a method to reduce the Inflammation‐plagued secondary cell death.

We found a substantial increase of astrocytic mitochondria in the primary neurons particularly in the HBOT‐preconditioned groups via Mitotracker labeling. Although there appears to be a natural transfer of mitochondria from astrocytes to neurons, as seen in the control cells grown under ambient cell culture condition, the presence of an injury to the neurons with exposure to TNF‐alpha or LPS significantly increased this astrocytic mitochondrial transfer (Figure 4). Additionally, HBOT preconditioning under ambient condition facilitated the astrocytic mitochondrial transfer when compared to the TNF‐alpha and LPS only groups (Figure 4). The combination of preconditioning and inflammatory insult further increased the astrocytic mitochondrial transfer (Figure 4), suggesting that neurons primed with a surplus of astrocyte mitochondria were better metabolically equipped to survive an inflammatory insult compared to neurons with only a small number of astrocytic mitochondria. That astrocytic mitochondria may be more resistant to insults than neuronal mitochondria partially supports the notion that astrocytes in general do not easily succumb to cell death after stroke compared to neurons.^35^, ^49^

**Figure 6:**
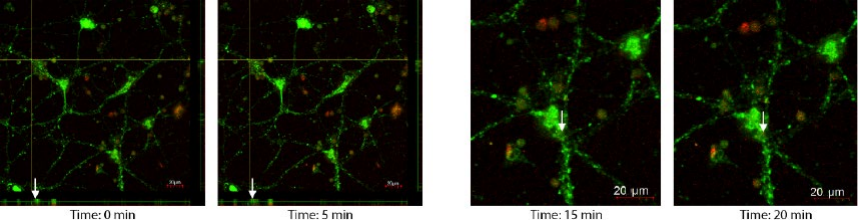
Live imaging of primary cortical neurons undergoing mitochondrial transfer. Rat E18 neuronal cells were harvested and seeded in poly‐D‐lysine coated (100 μg/mL) 6‐well plates at 1.5 × 10^6^ cells/well in Dulbecco's Modified Eagle Media high glucose with 1% antibiotic/antimycotic for 24 h. The media was changed every 3 d, and the cells were subcultured at 90% confluency as needed. Twenty‐four hours prior to the preconditioning, U87 astrocytes were stained with MitoTracker Deep Red FM (500 nmol/L) according to manufacturer's protocol and seeded into coculture inserts at 0.5 × 10^6^ cells/well. On the day of the experiment, rat E18 neuronal cells were stained with MitoTracker Green FM (200 nmol/L) according to manufacturer's protocol. The neuronal cells were then cocultured with U87 astrocytes for 3 h prior to Hyperbaric oxygen therapy (HBOT) administration. The cells were subjected to 70 min of HBOT at 2.5 ATA with 10 min pressurization and depressurization at a rate of 0.07 atm/min for a total of 90 min. Directly following HBOT treatment, the cocultured astrocytes were removed and the confocal z‐stacks live images were captured at 180×. Primary rat neuronal cell (PRNC) mitochondria: Green; Astrocyte mitochondria: Red. White arrows indicate the movement of astrocyte mitochondria into the PRNC during 5‐min intervals. The scale bar corresponds to 20 μm

Of equal translational importance, we also demonstrated that administration of HBOT at 2.5 ATA for 90 minutes was well tolerated as evidenced by maintained neuronal cell viability, highlighting the safety of the HBOT as a prophylactic treatment. Such initiation of HBOT before injury led to a significant neuroprotective effect (Figure 3). In addition, we found that the transfer of mitochondria occurred immediately within a short period (ie, 5 minutes) following HBOT (Figure 6) and persisted at least up to 20 minutes post‐HBOT (Figure 6). This observation of effective transfer of mitochondria even with acute HBOT prior to injury supports the use of a short bout of HBOT at low ATA as a powerful approach to induce neuroprotection, which circumvents reported adverse effects of prolonged HBOT at high ATA.^50^, ^51^

While not significantly detracting from our conclusions, there are limitations to this investigation. The live imaging obtained was conducted after completion of HBOT exposure, thereby preventing us from accurately pinpointing the onset of mitochondria transfers. Moreover, although a single bout of HBOT was shown here as safe and effective in promoting neuroprotection, repeated short HBOT exposures may provide more stable and long‐lasting functional outcomes considering the devastating neurological deficits acutely and chronically after a cerebrovascular event. The combination of pre‐ and postinjury HBOT will also warrant additional studies. In order to translate this preconditioning paradigm to the clinic, identification of a candidate population of individuals who are at an increased risk of cerebrovascular injury will be key to the successful enrollment of patients. The observed in vitro HBOT results definitely will require validation in in vivo disease models. In the end, testing a variety of HBOT conditions in clinically relevant models are critical to achieving the optimal safe and effective regimen of mitochondria transfer‐mediated neuroprotection.^52^ Recent studies have established successful protocols for testing HBOT in rodent models for both single and multiple treatments.^53^ Due to prior FDA‐approved indications, HBOT has an established infrastructure in clinics allowing for it to be quickly implemented once treatments are optimized for humans.^19^, ^20^

HBOT preconditioning poses as a prophylactic treatment for sequestration of inflammation, which is a pathological condition rampant in many cerebrovascular diseases. HBOT may be a leading alternative treatment for TBI and stroke modalities as steers away from invasive procedures such as exogenous cell transplantation following a major cerebrovascular event.^54^ Mitochondrial transfer from astrocytes to neurons is a potential primary mechanism of action of HBOT to conferring neuroprotective effects against inflammation. The ability to limit the severity of cerebrovascular injury in identified at‐risk individuals may reduce the health burden and socioeconomic load of these diseases on our healthcare system and economy.

## CONFLICT OF INTEREST

The authors declare no conflicts of interest.
